# PKC activation sensitizes basal-like breast cancer cell lines to Smac mimetics

**DOI:** 10.1038/cddiscovery.2016.2

**Published:** 2016-02-29

**Authors:** L Cornmark, C Holmgren, K Masoumi, C Larsson

**Affiliations:** 1 Department of Laboratory Medicine, Translational Cancer Research, Lund University, Lund, Sweden

## Abstract

There is a need for novel strategies to initiate cancer cell death. One approach is the use of Smac mimetics, which antagonize inhibitor of apoptosis proteins (IAPs). Recent studies have shown that combinations of Smac mimetics such as LBW242 or LCL161 in combination with chemotherapeutic agents increase cancer cell death. Here we show that the protein kinase C (PKC) activator TPA together with the Smac mimetic LBW242 induces cell death in two basal breast cancer cell lines (MDA-MB-468 and BT-549) that are resistant to Smac mimetic as single agent. Ten other LBW242-insensitive cancer cell lines were not influenced by the TPA+LBW242 combination. The TPA+LBW242 effect was suppressed by the PKC inhibitor GF109203X, indicating dependence on PKC enzymatic activity. The PKC effect was mediated via increased synthesis and release of TNF*α*, which can induce death in the presence of Smac mimetics. The cell death, coinciding with caspase-3 cleavage, was suppressed by caspase inhibition and preceded by the association of RIP1 with caspase-8, as seen in complex II formation. Smac mimetics, but not TPA, induced the non-canonical NF-*κ*B pathway in both MDA-MB-231 and MDA-MB-468 cells. Blocking the canonical NF-*κ*B pathway suppressed TPA induction of TNF*α* in MDA-MB-468 cells whereas isolated downregulation of either the canonical or non-canonical pathways did not abolish the Smac mimetic induction of the NF-*κ*B driven genes TNF*α* and BIRC3 in MDA-MB-231 cells although the absolute levels were suppressed. A combined downregulation of the canonical and non-canonical pathways further suppressed TNF*α* levels and inhibited Smac mimetic-mediated cell death. Our data suggest that in certain basal breast cancer cell lines co-treatment of TPA with a Smac mimetic induces cell death highlighting the potential of using these pathways as molecular targets for basal-like breast cancers.

## Introduction

Evasion of cell death is one important hallmark of cancer.^1,2^ Cell death comprises different subroutines^3,4^ with two main apoptotic pathways, the extrinsic and the intrinsic, as important examples.^5^ The extrinsic pathway is induced by death receptors (DRs) leading to the activation of caspase-8 whereas the intrinsic apoptotic pathway is initiated by cellular stress resulting in release of cytochrome *c* and second mitochondria-derived activator of caspase (Smac) from the mitochondria leading to activation of caspase-9. Both pathways converge in the activation of executioner caspases-3 and 7.^6,7^

One way to facilitate apoptosis induction and thereby circumvent the evasion of cell death by cancer cells is to mimic the function of Smac. Several small molecules mimicking Smac have been developed and some are under investigation in clinical trials.^8^

A Smac mimetic (SM) is thought to facilitate cell death by mimicking the antagonizing effect of Smac on inhibitor of apoptosis proteins (IAPs).^8^ Two IAPs, cellular IAP1 (cIAP1) and cIAP2, regulate tumor necrosis factor receptor 1 (TNFR1) signaling.^9^ TNFR1 activation can lead to extrinsic apoptotic signaling pathway. However, TNFR1 also induces NF-*κ*B signaling associated with survival and inflammation.^10^ The effect of the TNFR1 signal is determined by proteins that are available and recruited to the receptor.^11,12^ After ligand binding, cIAP1 and cIAP2 are recruited to the receptor and induce ubiquitination of other proteins, including receptor-interacting protein-1 (RIP1). Ubiquitination of RIP1 is necessary for the formation of complex I, an essential step for induction of NF-*κ*B signaling. SMs induce downregulation of cIAP1/2 thereby preventing complex I formation and NF-*κ*B signaling. Instead complex II, is formed. Complex II contains FADD, RIP1 and caspase-8 and leads to the activation of the latter and apoptosis.^11,12^ In addition, SM can facilitate cell death by blocking XIAP, a well-established caspase inhibitor.^13^

Some cancer cell lines die by SM treatment as single agent.^14–17^ The effect has been reported to be due to autocrine TNF*α* production, which induces cell death in the presence of SM.^16,17^ The TNF*α* production can be mediated by accumulation of NF-*κ*B-inducing kinase (NIK), followed by non-canonical NF-*κ*B signaling and TNF*α* transcription, which occur when cIAPs no longer ubiquitinate and target NIK for degradation.^17–19^ However, it is not completely clear what determines if a cell responds to a SM with TNF*α* production. It also raises the possibility that local induction of TNF*α* may be a way to make cancer cells susceptible to SM.

We previously found that the pro-apoptotic protein Smac and the protein kinase C (PKC) isoform PKC*δ* form a complex that is dissociated during cell death induction.^20^ Here we continue the investigation of Smac and PKC. We found that activation of PKC with subsequent synthesis and release of TNF*α* can overcome SM insensitivity in breast cancer cell lines of basal phenotype. The effect of TPA is dependent on the canonical NF-*κ*B pathway, whereas both the canonical and non-canonical pathways contribute to sensitivity to SM.

## Results

### PKC activation by TPA overcomes resistance to SM in MDA-MB-468 and BT-549 cells

**Figure 1:**
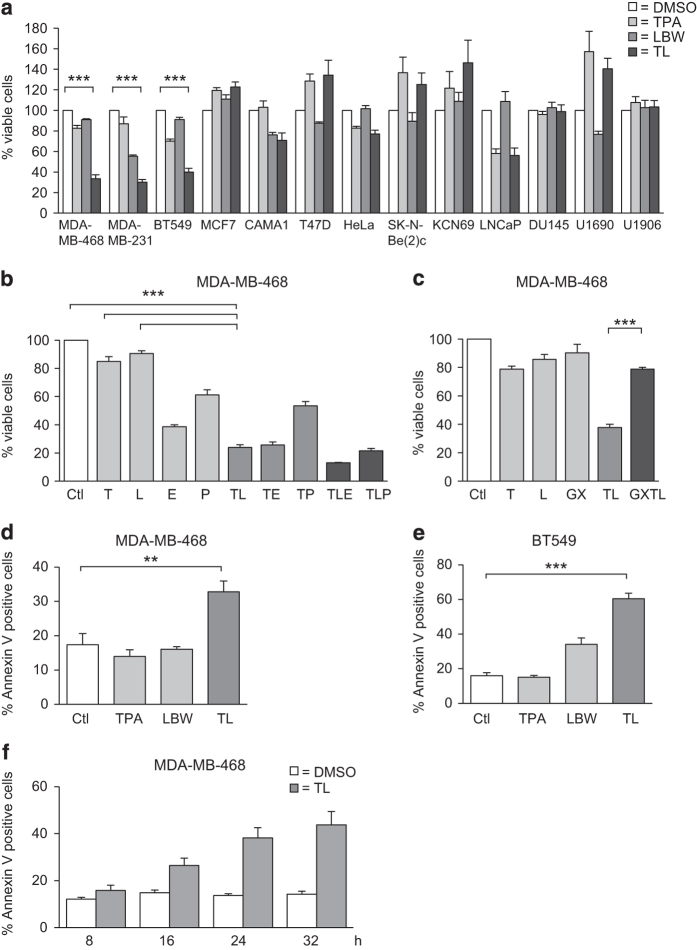
TPA overcomes resistance to LBW242 in MDA-MB-468 and BT-549 cells. (**a**) Different cancer cell lines were treated with 16 nM TPA, 20 *μ*M LBW242 or the combination of both (TL) for 30 h. (**b**) MDA-MB-468 cells were treated for 30 h with indicated combinations of 16 nM TPA (T), 20 *μ*M LBW242 (L), 100 *μ*M etoposide (E), 100 nM paclitaxel (P) or with corresponding volume of DMSO (Ctl) as control. (**c**) MDA-MB-468 cells were treated for 30 h with indicated combinations of 16 nM TPA, 20 *μ*M LBW242, 2 *μ*M GF109203X or with corresponding volume of DMSO as control. (**d**) MDA-MB-468 or (**e**) BT-549 cells were treated with 16 nM TPA and/or 20 *μ*M LBW242 for 16 h. (**f**) MDA-MB-468 cells were treated with 16 nM TPA and 20 *μ*M LBW242 for 8, 16, 24 and 32 h. Cell viability data in (**a**–**c**) were obtained with a WST-1 assay and are expressed as percentage of control cells. For (**d**–**f**) cell death was determined by flow cytometry using APC-conjugated annexin V. Data are mean±S.E.M. of *n*≥3 independent experiments; **P<0.01 and ***P<0.001.

To investigate a putative modulating effect of PKC on SM-induced death, we initially co-treated SM-sensitive MDA-MB-231^16,21^ and MDA-MB-468 cells, which are insensitive to SM treatment (Figure 1a), with the PKC activator TPA and the SM LBW242. TPA made MDA-MB-468 cells sensitive to SM and increased the effect of SM on MDA-MB-231 cells (Figure 1a). Only one more of nine additional cell lines tested, the BT-549 breast cancer cell line, showed a similar response (Figure 1a), except for LNCaP cells, which die upon treatment of TPA alone.^22^ The effects on MDA-MB-231 and MDA-MB-468 cells were the same with LCL161, which is another SM (Supplementary Figure 1). Because of limited availability of LBW242, LCL161 was used in some subsequent experiments.

We next evaluated the effects of TPA and SM together with the chemotherapeutic agents, etoposide and paclitaxel (Figure 1b). Both paclitaxel and etoposide alone suppressed cell viability and the effect was further enhanced by the addition of TPA and SM, indicating that the death-inducing effect can be even more pronounced with multiple agents. The TPA effect was blocked by the pan-PKC inhibitor GF109203X (Figure 1c), indicating that PKC activity is necessary for the effect.

Both MDA-MB-468 and BT-549 cells showed a significantly increased cell death, measured by an annexin V assay, upon treatment with the combination of TPA and SM (Figures 1d and e). Thus, the decreased cell viability is increased conceivably due to cell death.

After 16 h of treatment, an increase in cell death can be observed (Figure 1f), which is further increased with time.

### TPA in combination with SM induces complex II formation and caspase activation

**Figure 2:**
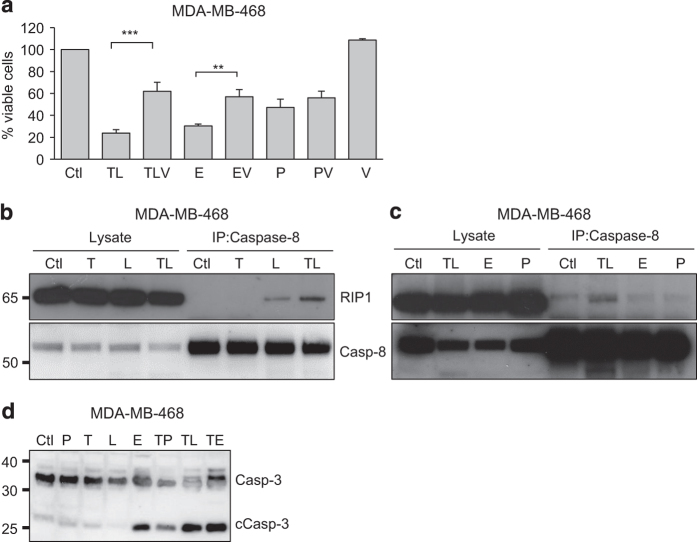
Combined treatment with TPA and LBW242 leads to caspase activation and complex II formation. (**a**) MDA-MB-468 cells were treated with indicated combinations of 16 nM TPA (T), 20 *μ*M LBW242 (L), 100 *μ*M etoposide (E), 100 nM paclitaxel (P) with or without 20 *μ*M of the pan-caspase inhibitor zVADfmk (V) or with corresponding volume of DMSO (Ctl) as control for 30 h. Cell viability was determined with a WST-1 assay and is expressed as percentage values obtained for DMSO-treated control cells. (**b**) MDA-MB-468 cells were treated with 16 nM TPA and/or 20 *μ*M LBW242 or (**c**) 16 nM TPA+20 *μ*M LBW242 (TL), 100 *μ*M etoposide or 100 nM paclitaxel for 16 h. All treatments were done in the presence of 20 *μ*M zVADfmk. Cell lysates were immunoprecipitated with caspase-8 antibodies and analyzed with western blot, using antibodies against RIP1 and caspase-8. (**d**) MDA-MB-468 cells treated with indicated combinations of 16 nM TPA, 20 *μ*M LBW242, 100 *μ*M etoposide, 100 nM paclitaxel or a DMSO control for 16 h were analyzed with western blot using antibodies against caspase-3. Data in (**a**) represent the mean±S.E.M. of *n*≥3 independent experiments; **P<0.01 and ***P<0.001. Blots in (**b**–**d**) are representatives of three independent experiments.

Next, we sought to investigate mechanisms mediating cell death by TPA+SM.^16,17^ Caspase inhibition resulted in a marked reduction in cell death induced by TPA+SM and by etoposide but had limited effects on paclitaxel-mediated death (Figure 2a). It has been proposed that SM enables the formation of complex II upon TNF*α* stimulation with subsequent activation of caspase-8.^16,17^ To evaluate the formation of complex II, we used an approach previously described^11,21^ where caspase-8, one of the constituents of complex II, is immunoprecipitated. When treating cells with TPA alone caspase-8 did not co-immunoprecipitate with RIP1. However, SM treatment led to co-immunoprecipitation of RIP1 and caspase-8, which was further strengthened by simultaneous incubation with TPA (Figure 2b). Neither etoposide nor paclitaxel induced a caspase-8-RIP1 complex (Figure 2c).

The combination of TPA and SM also resulted in the cleavage of caspase-3 whereas neither TPA nor SM alone had this ability (Figure 2d). TPA together with paclitaxel or etoposide only showed a weak capacity to affect caspase-3.

### TPA and SM-mediated cell death is TNF*α* dependent

**Figure 3:**
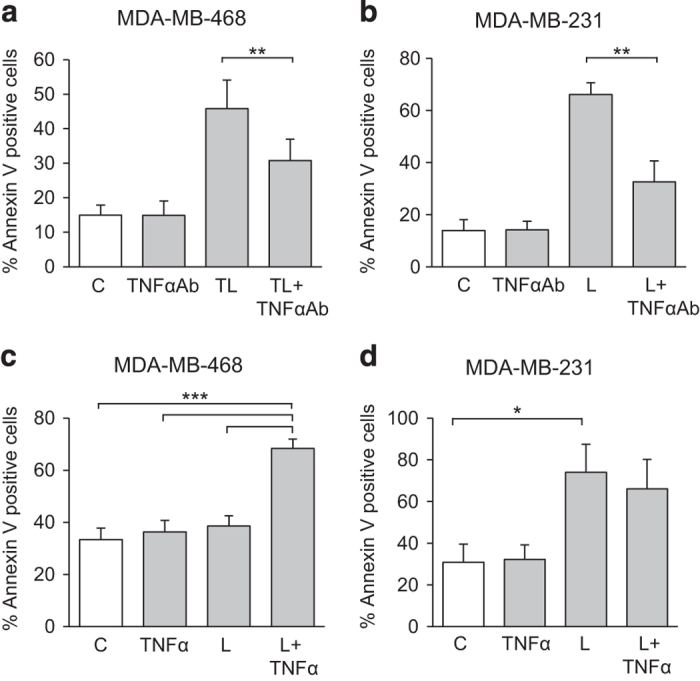
The cell death mediated by TPA and LBW242 is dependent on TNF*α*. Anti-human TNF*α* antibodies (2 *μ*g/ml) were added to both (**a**) MDA-MB-468 cells treated with 16 nM TPA and 20 *μ*M LBW242 as well as (**b**) MDA-MB-231 cells treated with 20 *μ*M LBW242 alone, for 16 h. (**c**) MDA-MB-468 and (**d**) MDA-MB-231 cells were treated for 16 h with 20 *μ*M LBW242 and/or 2 ng/ml TNF*α*. Cell death was determined by flow cytometry using APC-conjugated annexin V. All data are mean±S.E.M. of *n*≥3 independent experiments; *P<0.05, **P<0.01 and ***P<0.001.

Autocrine TNF*α* production has been reported to be important for SM-mediated cell death.^16,17^ We therefore examined if the cell death induced by TPA+SM is TNF*α* dependent as well. A TNF*α*-blocking antibody suppressed cell death induction both for MDA-MB-468 cells treated with TPA and SM as well as for MDA-MB-231 cells treated with SM alone (Figures 3a and b).

We next investigated whether TNF*α* is sufficient to induce cell death in combination with SM in MDA-MB-468 cells. TNF*α* alone had no effect but together with LBW242 a pronounced induction of cell death was seen (Figure 3c). For the SM-sensitive MDA-MB-231 cells no potentiating effect of TNF*α* could be seen (Figure 3d).

### TPA treatment leads to increased levels of TNF*α*

**Figure 4:**
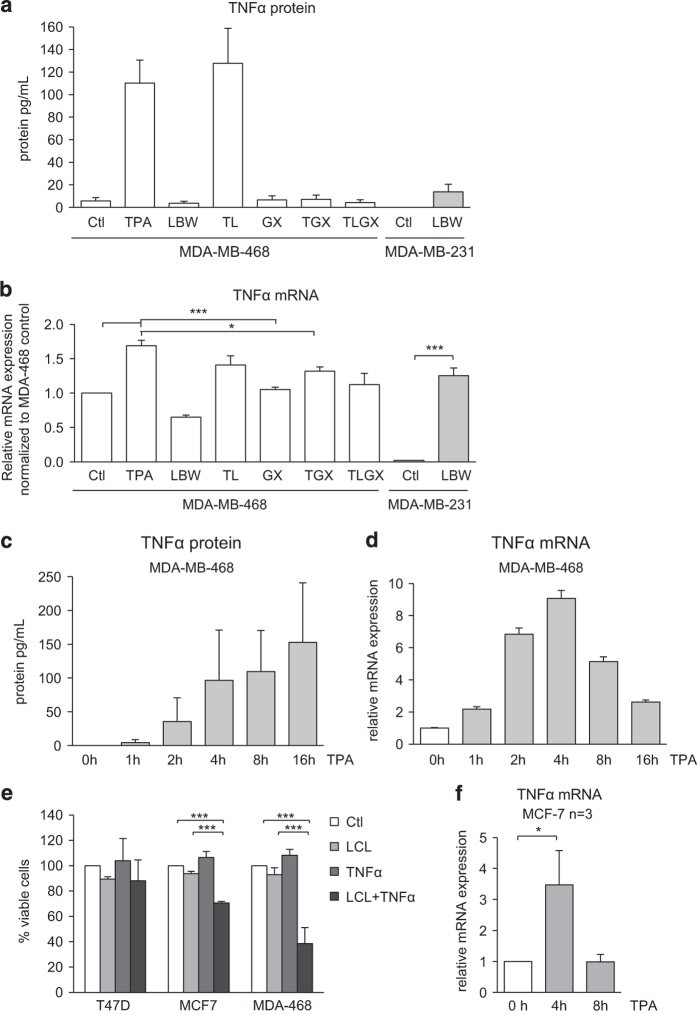
TNF*α* levels increase upon TPA treatment. (**a**) MDA-MB-468 and MDA-MB-231 cells were treated for 16 h with indicated combinations of 16 nM TPA (T), 20 *μ*M LBW242 (L) or 2 *μ*M GF109203X (GX). The concentration of TNF*α* protein in the cell culture medium was determined by ELISA. (**b**) TNF*α* mRNA levels were determined with qPCR and relative mRNA levels were normalized to control (Ctl) MDA-MB-468 cells. (**c** and **d**) MDA-MB-468 cells were treated with 16 nM TPA for 0, 1, 2, 4, 8 and 16 h, thereafter levels of TNF*α* protein in the cell culture medium (**c**) or mRNA of total cell lysate (**d**) was determined. (**e**) T47D, MCF-7 and MDA-MB-468 cells were seeded on a 96-well plate and treated with 2 ng/ml TNF*α*, 10 *μ*M LCL161 or the combination of both for 30 h. Cell viability data are obtained with a WST-1 assay and are expressed as percentage of control cells. (**f**) MCF-7 cells were treated with 16 nM TPA for 0, 4 or 8 h. Thereafter TNFa mRNA levels were determined with qPCR. All data are mean±S.E.M. of *n*≥3 independent experiments; *P<0.05 and ***P<0.001.

To investigate if TPA treatment results in TNF*α* production, we investigated TNF*α* levels in cell culture medium. TPA induced higher TNF*α* protein concentrations in the cell culture medium of MDA-MB-468 cells whereas SM had no effect, neither in the absence nor presence of TPA (Figure 4a). GF109203X abolished the effect of TPA. Contrasting MDA-MB-468 cells, SM alone resulted in increased TNF*α* levels in MDA-MB-231 cells (Figure 4a).

In MDA-MB-468 cells, TPA treatment also caused a modest (69%) increase in TNF*α* mRNA levels and SM had no effect. On the other hand, TNF*α* mRNA levels in SM-treated MDA-MB-231 cells were markedly increased compared to basal levels (Figure 4b).

TPA led to a more than 19-fold elevation of TNF*α* protein concentration but only a 69% increase in TNF*α* mRNA levels in MDA-MB-468 cells. To analyze this discrepancy, the effect of TPA stimulation at different time points was investigated. TNF*α* protein levels gradually increased during the first 16 h (Figure 4c), whereas TNF*α* mRNA levels preceded protein changes and peaked after 4 h of TPA treatment (Figure 4d). This conceivably explains the discrepancies between protein and mRNA levels after 16 h of treatment.

According to cell viability results (Figure 1a), most cell lines do not respond with decreased cell viability when combining a SM with TPA. This could potentially be due to an inability of TPA to induce TNF*α* or a resistance to the combination of SM with TNF*α*. To assess these hypotheses, T47D and MCF-7 cells were treated with SM and TNF*α* followed by analysis of cell viability (Figure 4e). T47D cells were insensitive to this combination conceivably explaining the insensitivity to TPA and SM treatment. Although some cell death was induced in MCF-7 cells by the treatment the effect was not as pronounced as for MDA-MB-468 cells. Furthermore, TPA treatment of MCF-7 cells resulted in TNF*α* mRNA production but with lower and faster declining levels than in MDA-MB-468 cells (Figures 4d and f). Thus, the insensitivity of MCF-7 cells to the combination of TPA and SM may depend on both a lower sensitivity to the combination of TNF*α* and SM, and a smaller induction of TNF*α* by TPA.

### Global gene expression of SM-treated sensitive and insensitive cell lines

**Figure 5:**
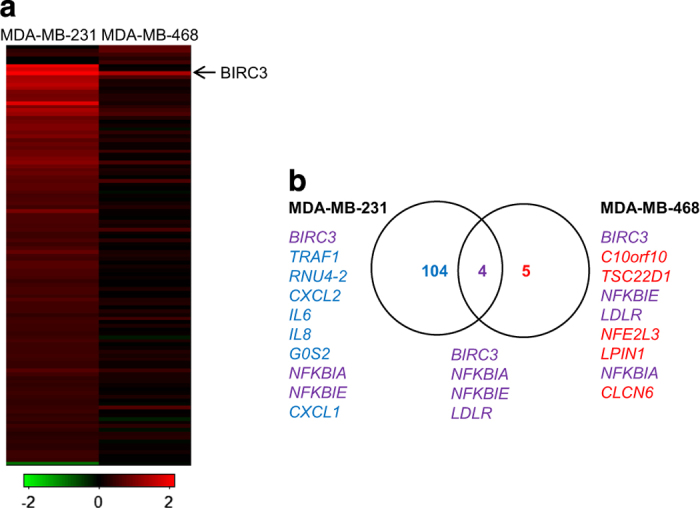
(**a**) Heatmap visualizing SM-induced fold change in gene expression of genes with a *q*-value of 0% for MDA-MB-231 (left) and MDA-MB-468 (right). The color code indicates log2 fold change induced by LBW242. (**b**) Venn diagram displaying the significantly differentially expressed genes, retrieved from the microarray and the subsequent SAM analysis. The genes indicated in the diagram represents all (MDA-MB-468) genes or the top ten (MDA-MB-231) with the highest log2 fold change, in descending order, induced by LBW242 and with a *q*-value=0% (Supplementary Table 1), in MDA-MB-231 (gene symbol in blue), MDA-MB-468 (gene symbol in red) or in both cell lines (gene symbol in purple).

To further delineate mechanisms mediating TNF*α* expression, we turned to comparison of the response to SM and analyzed changes in global gene expression following SM treatment of MDA-MB-231 and MDA-MB-468 cells. The most apparent finding was that SM-treated MDA-MB-231 cells had a substantially higher number of differentially expressed genes (108 genes) compared to the SM-treated MDA-MB-468 cells (nine genes), of which four were affected in both cell lines, when applying a *q*-value of 0% as a cutoff. All differentially expressed genes, except for *OSR1* in MDA-MB-231 cells were expressed at higher levels following SM treatment (Figures 5a and b). Many of the SM-induced genes are NF-*κ*B target genes, such as BIRC3, CXCL1, IRAK2, TNF and TRAF1^23^ (Supplementary Table 1), which is further supported by a pathway analysis (Supplementary Figure 3).

### Effects of SM on NF-*κ*B signaling in sensitive and insensitive cells

**Figure 6:**
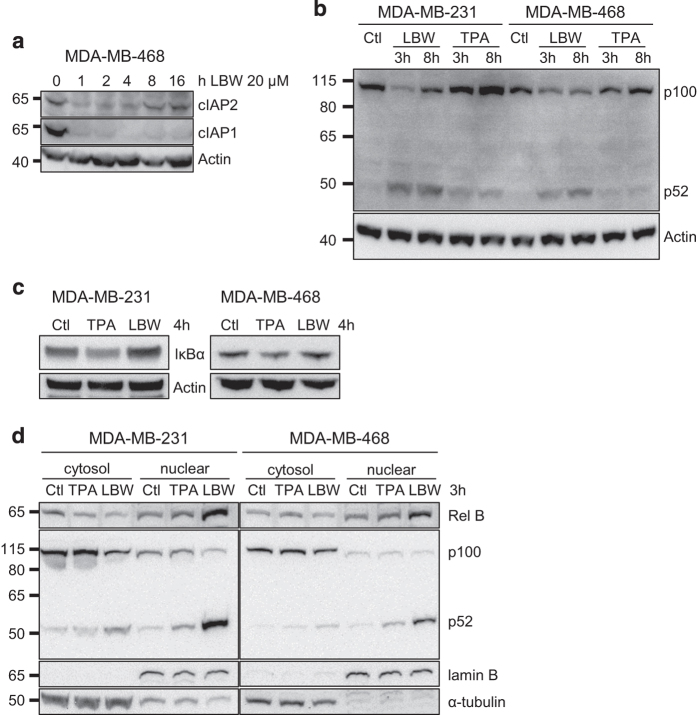
LBW242 activates the non-canonical NF-*κ*B pathway in both MDA-MB-231 and MDA-MB-468 cells. (**a**) MDA-MB-468 cells were treated with 20 *μ*M LBW242 for indicated time periods. Cell lysates were analyzed with western blot using antibodies targeting cIAP1 and cIAP2. (**b**) MDA-MB-231 and MDA-MB-468 cells were treated with 16 nM TPA or 20 *μ*M LBW242 for 3 or 8 h. Levels of p100 and p52 were analyzed with western blot. (**c**) MDA-MB-231 and MDA-MB-468 cells were treated with 16 nM TPA or 20 *μ*M LBW242 for 4 h. Levels of I*κ*B*α* were analyzed with western blot. In (**a**–**c**) actin was used as a loading control. (**d**) Cytosolic- and nuclear fractions from MDA-MB-231 and MDA-MB-468 cells treated with 16 nM TPA or 20 *μ*M LBW242 for 3 h were analyzed for Rel B, p100 and p52 with western blot. Lamin B and *α*-tubulin are included as markers of nuclear and cytosolic fractions, respectively. All blots are representatives of three independent experiments.

To investigate why SM treatment leads to a more limited effect on gene expression in MDA-MB-468 than in MDA-MB-231 cells, molecular pathways known to be influenced by SM were analyzed. Exposure to SM resulted in an initial degradation of both cIAPs in MDA-MB-468 cells that was sustained for cIAP1 but transient for cIAP2 (Figure 6a). The same pattern has been described in MDA-MB-231 cells.^21^ Thus, this effect is similar in the two cell lines.

One possible outcome of cIAP1/2 degradation is the stabilization of NIK and subsequent processing of p100 to p52, which is indicative of non-canonical NF-*κ*B signaling. SM treatment led to processing of p100 to p52 in both cell lines after 3 h (Figure 6b). TPA did not result in p100 processing in either cell line. Instead we found decreased levels of I*κ*B*α* protein following TPA treatment (Figure 6c), whereas SM had no effect here. This suggests that primarily the canonical NF-*κ*B pathway is involved in TPA-mediated TNF*α* production.

To assess whether the different sensitivity to SM may be related to a differential ability for the processed p52 fragment to enter the nucleus, nuclear and cytosolic fractions of TPA or SM-treated MDA-MB-231 and MDA-MB-468 cells were prepared. After 3 h of LBW242 treatment, p52 as well as the heterodimer Rel B appeared in the nuclear fraction of both cell lines (Figure 6d). Corroborating the results seen in Figure 6b, TPA did not evoke nuclear localization of p52 or Rel B (Figure 6d).

### Effects of suppressing non-canonical or canonical NF-*κ*B signaling

**Figure 7:**
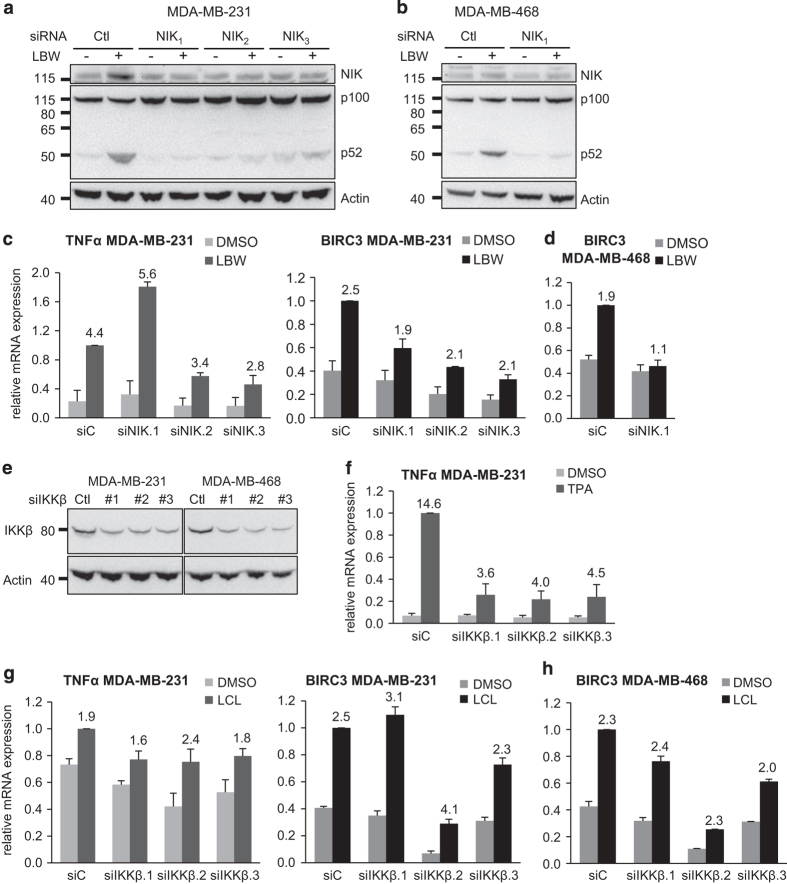
Individual suppression of non-canonical and canonical NF-*κ*B signaling. (**a**) MDA-MB-231 cells were transfected with three different siRNA oligos (**b**) and MDA-MB-468 were transfected with one of the siRNA oligos targeting NIK. Both cell lines were treated with 20 *μ*M LBW242 for 3 h. NIK expression and processing of p100 to p52 was evaluated by western blot, one representative blot out of three independent experiments is shown. Actin is used as a loading control. (**c** and **d**) The mRNA levels of TNF*α* and BIRC3 were determined by qPCR. (**e**) MDA-MB-231 and MDA-MB-468 cells were transfected with three different siRNA oligos targeting IKK*β*, protein expression was evaluated with western blot. (**f**) Transfected MDA-MB-231 cells were treated with 16 nM TPA for 3 h and TNF*α* mRNA levels were analyzed with qPCR. (**g** and **h**) Transfected cells were treated with 10 *μ*M LCL161 for 3 h. TNF*α* and BIRC3 mRNA levels were evaluated with qPCR. The numbers above each bar indicate the fold change in mRNA levels between treated and untreated samples for each siRNA. All qPCR results are mean±S.E.M. of *n*=3 independent experiments.

Both MDA-MB-231 and MDA-MB-468 cell lines respond to SM with p100 processing and nuclear p52 translocation (Figures 6b and d), which indicates activation of the non-canonical NF-*κ*B pathway in both cell lines. However, SM evokes substantial changes in global gene expression including TNF*α* only in MDA-MB-231 cells (Figures 4a and b; Supplementary Table 1). These facts raise the question if non-canonical NF-*κ*B signaling is the actual or sole mediator of TNF*α* induction. Therefore, we sought to investigate SM-induced TNF*α* expression when blocking either the non-canonical or the canonical pathway. NIK, an initiator of the non-canonical pathway, was downregulated with three different siRNA oligonucleotides. SM treatment induced NIK accumulation in MDA-MB-231 cells transfected with control siRNA, but this was suppressed in cells transfected with siRNA targeting NIK (Figure 7a). The same was seen for p100 processing to p52, which was essentially abolished in SM-treated cells transfected with NIK siRNA indicating a functional termination of the non-canonical NF-*κ*B pathway. The effects were similar using both the LBW242 and the LCL161 SM (Figure 7a; Supplementary Figure 2a). Similar results were obtained in MDA-MB-468 cells (Figure 7b).

With siNIK as a tool to block the non-canonical NF-*κ*B pathway, we investigated the SM-induced expression of BIRC3, the most upregulated gene in the microarray analysis, and TNF*α* (Figure 7c). In siNIK-transfected and SM-treated MDA-MB-231 cells, a decrease in SM-stimulated absolute levels of TNF*α* and BIRC3 mRNA was observed except for one siRNA in the case of TNF*α*. However, the fold change in TNF*α* and BIRC3 mRNA induced by SM was not as influenced by siRNA treatment (Figure 7c). This contrasts MDA-MB-468 cells, where downregulation of NIK completely suppressed SM-induced increases in BIRC3 mRNA levels (Figure 7d).

Thus, SM induction of TNF*α* expression can proceed to some extent in MDA-MB-231 cells even if the non-canonical NF-*κ*B pathway is blocked (Figure 7c). This opens up for a possible role of other pathways. Therefore, we investigated the involvement of the canonical NF-*κ*B pathway using siRNA targeting IKK*β* (Figure 7e). Downregulating IKK*β* with three different siRNAs, all resulted in a strong reduction of TPA-induced TNF*α* mRNA expression (Figure 7f), indicating a functional suppression of the canonical NF-*κ*B signal. However, downregulation of IKK*β* did not have substantial effects on the SM-induced fold changes in TNF*α* or BIRC3 mRNA levels in MDA-MB-231 cells (Figure 7g) although the absolute levels were suppressed in most cases. The pattern was the same for BIRC3 mRNA expression in MDA-MB-468 cells (Figure 7h).

### A combined role for the canonical and non-canonical NF-*κ*B pathways

**Figure 8:**
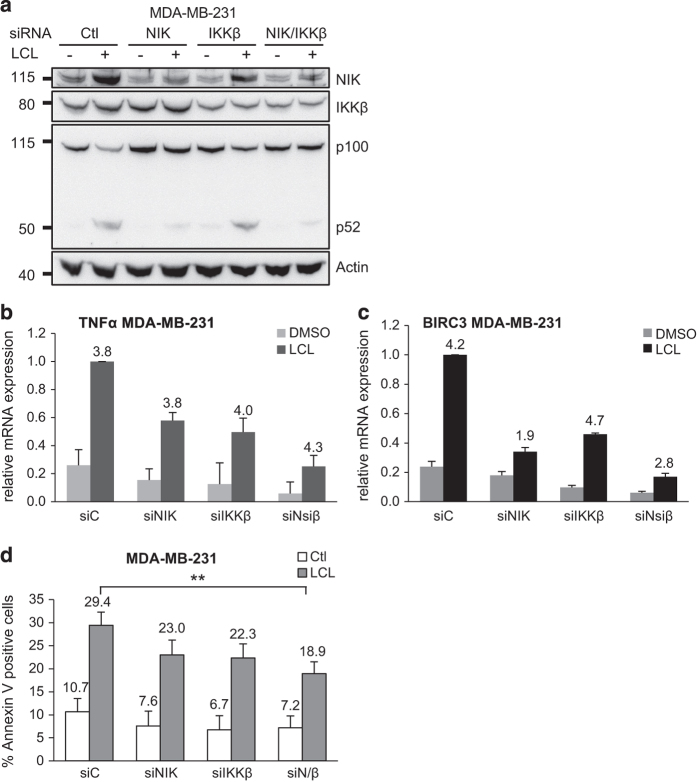
Combined suppression of the canonical and non-canonical NF-*κ*B pathways. MDA-MB-231 cells were transfected with siRNA oligos targeting NIK, IKK*β* or the combination of both for 48 h and treated with 10 *μ*M LCL161 for 3 h. (**a**) NIK, IKK*β*, p100 and p52 protein expression was evaluated with western blot, and actin was used as a loading control. (**b, c**) TNF*α* and BIRC3 mRNA levels were analyzed with qPCR. The numbers above each bar indicate the fold change of mRNA levels between treated and untreated sample for each siRNA. (**d**) Cell death was determined by flow cytometry using APC-conjugated annexin V. All results represent the mean±S.E.M. of *n*=4 independent experiments; **P<0.01.

To investigate the possibility of compensatory mechanisms, we inhibited both the canonical and the non-canonical NF-*κ*B pathways with siRNAs targeting NIK and IKK*β*. The absolute levels of SM-induced TNF*α* and BIRC3 mRNA expression in MDA-MB-231 cells were suppressed upon downregulation of each protein alone with an even stronger effect after downregulation of both proteins in combination (Figures 8a–c). However, the SM-induced fold change still persisted following combined knockdown of NIK and IKK*β* even though the absolute TNF*α* mRNA levels were low, resembling levels of control cells (Figure 8b). The pattern was the same for BIRC3 (Figure 8c). In line with effects on TNF*α*, the SM-induced cell death was suppressed following treatment with siRNA targeting IKK*β* or NIK (Figure 8d). Combined knockdown of both IKK*β* and NIK resulted in a significant decrease in cell death whereas knockdown of only one pathway led to a smaller effect. The results indicate that both the canonical and the non-canonical NF-*κ*B pathways contribute to SM-induced TNF*α* levels and cell death.

## Discussion

Deregulation of cell death is an important contribution to cancer development and may also confer resistance to chemotherapy and radiotherapy. Therefore, promoting cell death induction is one potential strategy for cancer therapy.

One way to promote cell death is by mimicking the function of the pro-apoptotic protein Smac with SM. However, although some cells respond with cell death, most cell types are resistant to SM as single agents. In this report, we show that the PKC activator TPA induces sensitivity to SM in certain basal breast cancer cells and shed light on the differences in SM-induced signaling between sensitive and insensitive cells.

Current understanding indicates that SM-induced cell death involves autocrine TNF*α* production,^16,17^ although SM-induced cell death independently of TNF*α* has been reported.^13,14,24^ Our data indicate that TPA promotes sensitivity to SM by initiating the production of TNF*α*, since TPA induced a *de novo* production of TNF*α* with increased TNF*α* mRNA and protein levels (Figure 4). Furthermore, the TPA effect was blocked by TNF*α* antibodies, and could be mimicked by addition of TNF*α* (Figures 3a–d). Thus, induction of local production of TNF*α* may be one way to render cancer cells sensitive to SM, a notion in line with the report that pathogen mimetics generating a cytokine milieu consisting of IFN*β*, TNF*α* and/or TRAIL sensitizes cancer cells to SM.^25^

The fact that PKC activation can induce TNF*α* production in many cell lines^26–29^ and the proposed dependency on TNF*α* for SM-induced death,^16,17^ raise the question why most cell lines were insensitive to the TPA+SM combination (Figure 1a). In fact, only triple-negative breast cancer cell lines were sensitive to this treatment. There are several putative explanations for this, one being that TPA may not lead to sufficiently high TNF*α* levels in all cell types. One example of this is MCF-7 cells where TPA induces TNF*α* expression but with shorter duration and at lower levels than in MDA-MB-468 cells (Figure 4f). Another possibility is that the cell does not respond to TNF*α*. This was the case for T47D cells, which were insensitive to the combination of SM and TNF*α* (Figure 4e). Insensitivity to the combination of SM and TNF*α* has also been reported for mesothelioma cells. This was reported to be due to the caspase inhibitory actions exerted by the caspase-8 inhibitor FLICE-like inhibitory protein (cFLIP).^30^

TNF*α* is a target gene of the NF-*κ*B signaling pathway^31^ and TPA has been shown to induce NF-*κ*B activation in several cell lines.^26,28^ More recent studies indicate PKC*δ* and/or PKC*ε* isoforms as mediators of TPA-induced NF-*κ*B activation.^27,29^ Our data show that TPA treatment of MDA-MB-231 and MDA-MB-468 cells results in decreased I*κ*B*α* levels, indicative of canonical NF-*κ*B signaling (Figure 5c), and that downregulation of the canonical pathway (Figure 7f) suppresses the TNF*α* induction. It is therefore conceivable that *de novo* synthesis of TNF*α*, via the canonical NF-*κ*B pathway, is the primary effect of TPA treatment in MDA-MB-468 breast cancer cells.

Contrasting the effects of TPA, it has been implied that SM induces TNF*α* production via the non-canonical NF-*κ*B pathway.^16–18^ This pathway is mediated through activation of NIK. SM treatment can lead to NIK stabilization and activation due to decreased levels of cIAP1 and cIAP2 that ubiquitinate NIK, causing its degradation.^16–18^ Here we show that SM leads to decreased levels of both cIAP1 and cIAP2 in MDA-MB-468 cells (Figure 5a) as has previously been described for MDA-MB-231 cells.^21^ The subsequent processing of p100 to p52 was seen for both cell lines (Figure 5b). Thus, the non-canonical NF-*κ*B signaling pathway is activated by SM in both cell lines but results in TNF*α* expression only in MDA-MB-231 cells. This may suggest that non-canonical NF-*κ*B signaling is not sufficient for TNF*α* induction. Our microarray data further indicate that non-canonical signaling does not elicit major changes in gene expression in MDA-MB-468 cells since few genes were upregulated upon SM treatment contrasting the effect in MDA-MB-231 cells. However, both MDA-MB-468 and MDA-MB-231 cell lines showed a substantial increase in BIRC3 expression following SM treatment indicating similar responses to non-canonical NF-*κ*B signaling for this gene.

The fact that the non-canonical pathway is not sufficient for SM-mediated TNF*α* production in MDA-MB-468 raises the question whether other pathways are needed. There is substantial support for a role of NF-*κ*B signaling in SM-induced TNF*α* expression.^32,33^ Here we used siRNA to inhibit the canonical and the non-canonical NF-*κ*B pathways separately or in combination. Blocking either pathway in the sensitive MDA-MB-231 cell line resulted in lower absolute levels of the NF-*κ*B target genes, TNF*α* and BIRC3 (Figures 7c and g). However, the ability of SM to induce a relative increase still remained. To suppress SM-stimulated TNF*α* and BIRC3 levels down to those of untreated control and to mediate a significant decrease in cell death, inhibition of both pathways were required (Figures 8b–d). However, even when both NF-*κ*B pathways are suppressed there is still a relative increase in TNF*α* mRNA upon SM treatment suggesting that additional IAP-regulated mechanisms may be involved. There are several examples of NF-*κ*B-independent roles for IAPs, such as facilitation of Myc-promoted proliferation by ubiquitinating the Myc-negative regulator Max-dimerization protein-1 (Mad1),^34^ control of cyclin transcription by regulation of E2F1^35^ and the findings that cIAP1 and XIAP affect morphology of cells by acting on Rho GTPases.^36,37^

Other potential mechanisms are suggested in studies identifying mechanisms of SM resistance, such as *in vivo*-selected SM-resistant MDA-MB-231 sublines where lower levels of the leucine-rich repeats and immunoglobulin-like domains 1 (LRIG1) were found. LRIG1 depletion also attenuated production of and influenced the sensitivity to TNF*α*.^38^ Furthermore, the interferon regulatory factor 1 (IRF1) was shown to be important for SM-mediated TNF*α* induction and SM sensitivity.^39^ Another report showed that overexpression of USP11 led to stabilization of cIAP2 and resistance to SM.^40^

Taken together, this paper further strengthens the importance of TNF*α* in SM-mediated cell death and demonstrates that a simultaneous induction of TNF*α* may be one strategy to increase SM sensitivity. At least for basal-like breast cancer cells, this can potentially be achieved by PKC activation. We also provide indications that induction of the non-canonical NF-*κ*B pathway may not be sufficient and not the sole mediator of SM-induced TNF*α* production. It is conceivable that other mechanisms are necessary to occur.

## Materials and Methods

### Materials and cell culture

All cell lines were from American Type Culture Collection (ATCC, Manassas, VA, USA). All media and supplements were from Thermo Scientific (Waltham, MA, USA) unless stated otherwise. Cells were grown in RPMI-1640 except for HeLa and Cama-1 grown in DMEM low glucose, and KCN-69 and SK-N-BE2C grown in MEM/EBSS. All media were supplemented with 10% fetal bovine serum (Biosera, Boussens, France), 100 IU/ml penicillin, and 100 *μ*g/ml streptomycin. In addition, MDA-MB-468 and MDA-MB-321 media were supplemented with 1 mM sodium pyruvate; BT-549 medium was supplemented with 0.023 IU/ml Actrapid Penfill insulin (Novo Nordisk AS, Bagsvaerd, Denmark); and Cama-1 medium was supplemented with 1% MEM non-essential amino acids.

When indicated, cells were treated with etoposide, paclitaxel, TNF-*α*, TPA (all Sigma-Aldrich, St. Louis, MO, USA), anti-hTNF-*α*-hIgG2 (InvivoGen, Toulouse, France), GF109203X (Calbiochem, San Diego, CA, USA) and zVADfmk (Enzo Life Sciences, Farmingdale, NY, USA). LBW242 and LCL161 were kindly provided by Novartis (Basel, Switzerland).

### Transfections

For siRNA transfections, cells were seeded at a density of 1 000 000–1 500 000 cells per 100-mm cell culture dish in 8 ml complete medium without antibiotics. After 24 h, cells were incubated for 48 h with 2 *μ*l/ml Lipofectamine 2000 (Life Technologies—Invitrogen, Waltham, MA, USA) and 40 nM siRNA in Opti-MEM (Life Technologies—Gibco, Waltham, MA, USA) and complete medium without antibiotics according to the supplier’s protocol.

### Annexin V-staining

Cells were seeded at a density of 2 000 000 cells per 100-mm cell culture dish (1 000 000 cells for siRNA-transfected samples) in 8 ml complete medium. Twenty-four hours later the medium was changed and cells were treated with indicated compounds. Cells were stained with annexin V-APC (BD Pharmingen, Franklin Lakes, NJ, USA) and analyzed with FACSCalibur or BD FACSVerse (both BD Biosciences, Franklin Lakes, NJ, USA). In total, 10 000 events were acquired on the FL-4 channel for the annexin V-APC signal. Sample acquisition and analyses were performed with CellQuest software or BD FACSuite software (both BD Biosciences).

### Analysis of cell viability

Cells were seeded in 100 *μ*l complete medium at a density of, depending on cell line, 6000–12 000 cells per well in 96-well culture plates. After 24 h, the medium was changed and cells were treated with indicated compounds for 30 h of which the last 4 h were in combination with 10 *μ*l WST-1 (Roche, Penzberg, Germany). The amount of viable cells was thereafter assessed by measuring the conversion of WST-1 to a water-soluble formazan dye. Absorbance (450 nm) was measured by the Synergy 2 Microplate reader (BioTek, Winooski, VT, USA) and analyzed with the Gen5 Reader Control and Data Analysis software (BioTek).

### Analysis of complex II formation

For complex II immunoprecipitations, cells were treated with indicated agents in the presence of 20 *μ*M zVADfmk to enable uncleaved caspase-8 immunoprecipitation as previously described.^21^ Cells were washed once and collected in ice-cold PBS and centrifuged before adding 500 *μ*l kit lysis buffer (Miltenyi Biotec, Bergisch Gladbach, Germany) containing 40 *μ*l/ml complete protease inhibitors (Roche Applied Science, Penzberg, Germany). Lysates were cleared by centrifugation at 14 000×*g* for 10 min. Immunoprecipitations were performed using caspase-8 antibodies (1 : 75, Cell Signaling 9746, Danvers, MA, USA) together with μMACS Protein G MicroBeads (Miltenyi Biotec).

Immunoprecipitates were added to MACS Separation Columns (Miltenyi Biotec), washed in kit buffers and proteins were eluted with heated sample buffer containing the reducing agent dithiothreitol.

### Western blot

Cells were washed in ice-cold PBS and lysed in RIPA buffer (10 mM Tris-HCl (pH 7.2), 160 mM NaCl, 1% Triton X-100 (Sigma-Aldrich), 1% sodium deoxycholate, 0.1% sodium dodecyl sulfate, 1 mM EDTA and 1 mM EGTA) containing 40 *μ*l/ml complete protease inhibitors (Roche Applied Science). Lysates were cleared by centrifugation at 14 000×*g* for 10 min. Equal amounts of proteins were electrophoretically separated on 10% NuPAGE Novex Bis-Tris gels (Life Technologies—Invitrogen) and transferred to polyvinylidene difluoride membranes (Millipore, Billerica, MA, USA). Membranes were blocked with 5% non-fat milk in PBS, and probed with antibodies toward actin (1 : 2000, MP Biomedicals 691001, Santa Ana, CA, USA and 1 : 1000 sc-81178), caspase-3 (1 : 500, Pharmingen 67341A), caspase-8 (1 : 500, Cell Signaling 97465), cIAP1 (1 : 200, R&D AF8181, Minneapolis, MN, USA), cIAP2 (1 : 300 NB110-57030), p52/p100 (1 : 300, Cell Signaling #4882), Rel B (1 : 300 sc-48366), p65 (1 : 400 sc-372), *α*-tubulin (1 : 400 sc-5286), lamin B (1 : 400 sc-6216) and RIP1 (1 : 300 BD Trans Lab 610458, Franklin Lakes, NJ, USA), followed by incubation with a horseradish peroxidase-labeled secondary antibody (1 : 5000 GE Healthcare, Little Chalfont, UK). The chemiluminescence was detected with a charge-coupled device camera (Fujifilm, Minato, Japan).

### Analysis of TNF*α* mRNA levels

Total RNA was extracted with the RNeasy kit (Qiagen, Hilden, Germany) and potential DNA contamination was eliminated with the RQ1 RNase-free DNase (Promega, Madison, WI, USA). In total, 2 *μ*g of RNA was used for cDNA synthesis with MultiScribe Reverse Transcriptase (Applied Biosystems, Foster City, CA, USA). The cDNA was thereafter amplified by qPCR for evaluation of relative mRNA expression levels in an Applied Biosystems 7300 real-time quantitative PCR system using the SYBR Green PCR Master Mix (Applied Biosystems). All primer pairs were from Invitrogen Life Sciences (Waltham, MA, USA), designed with the Primer Express software (Applied Biosystems; forward TNF: 5′-GCAGGTCTACTTTGGGATCATTG-3′, reverse TNF: 5′-GCGTTTGGGAAGGTTGGA-3′). The mRNA expression data were normalized to three reference genes (*SDHA*, *UBC* and *YWHAZ*) as described before.^41^ For relative quantification of gene expression, the comparative Ct method was applied.^42^

### Enzyme-linked immunosorbent assay (ELISA)

TNF*α* protein levels in the cell culture media were determined using the human TNF alpha ELISA kit (Thermo Scientific) according to the manufacturer’s protocol. Sample diluent was added to an anti-human TNF*α* precoated 96-well strip plate along with cell culture media, from cells with indicated treatment, or with standard and incubated for 1 h. After rinsing the plate with washing buffer, biotinylated antibodies were added, incubated for 1 h and washed. Streptavidin-HRP reagent was added for 30 min, whereafter the plate was washed before incubation with TMB substrate solution for 30 min in the dark. Following addition of stop solution, absorbance (at 450 and 550 nm) was measured in a Synergy 2 Microplate reader (BioTeck) and analyzed with the Gen5 Reader Control and Data Analysis software (BioTek).

### Nuclear fractionation

Cell fractionation protocol was adapted after Kasperczyk *et al*.^43^ Briefly, the collected and pelleted cells on ice were resuspended in a low-salt buffer (10 mM HEPES-OH (pH 7.9), 1.5 mM MgCl_2_ and 10 mM KCl) and incubated at 4 °C for 10 min. Thereafter, NP-40 with a final concentration of 1% was added and the sample was vigorously mixed before centrifugation at 1000×*g* for 5 min at 4 °C. The supernatant was centrifuged at 12 500×*g* for 10 min at 4 °C and the resulting supernatant was collected as the cytosol fraction. To retrieve the nuclear fraction, the first pellet was dissolved in a high-salt buffer (20 mM HEPES-OH (pH 7.9), 420 mM NaCl, 1.5 mM MgCl_2_, 0.2 mM EDTA and 25% glycerol) incubated for 15 min at 4 °C during which time the pellet was vortexed periodically before centrifugation at 12 500×*g* for 10 min at 4 °C. The resulting supernatant was collected as the nuclear fraction. Both low- and high-salt buffers were supplemented with a protease- and phosphatase inhibitor cocktail: 40 *μ*l/ml complete protease inhibitors (Roche Applied Science), 1 mM sodium-orthovanadate (Na_3_VO_4_) and 50 mM *β*-glycerophosphate (both from Sigma-Aldrich) before use.

### Microarray analysis

RNA was extracted from MDA-MB-231 and MDA-MB-468 cells after 4 h of treatment with LBW242 or DMSO using the RNeasy mini kit (Qiagen). Total gene expression analysis was performed by the SCIBLU facility in Lund using the DirectHyb HumanHT-12 v4.0 chip (Illumina, San Diego, CA, USA). Results were normalized using quantile normalization. The significance analysis of microarrays (SAM)^44^ analysis was performed to identify significantly differentially expressed genes in LBW242-treated cells *versus* control cells. Pathway analysis was performed using the MetaCore web-based program (MetaCore pathway analysis software) by SCIBLU.

### Statistical analysis

Statistical analyses were performed using IBM SPSS Statistics 22 and one-way ANOVA followed by Duncan’s multiple range test.

## Abbreviations

PKC: protein kinase c
Smac: second mitochondria-derived activator of caspases
IAP: inhibitor of apoptosis protein
TPA: 12-*O*-tetradecanoylphorbol-13-acetate
TNF*α*: tumor necrosis factor alpha
RIP1: receptor-interacting protein kinase-1
NF-*κ*B: nuclear factor kappa-light-chain enhancer of activated B cells
BIRC3: baculoviral IAP repeat-containing 3
DR: death receptor
SM: Smac mimetic
cIAP: cellular IAP
TNFR1: tumor necrosis factor receptor-1
FADD: fas-associated protein with death domain
XIAP: X-linked IAP
NIK: NF-*κ*B-inducing kinase
IKK*β*: I-kappa-B kinase-*β*
IFN*β*: interferon beta
TRAIL: TNF-related apoptosis-inducing ligand
cFLIP: cellular FLICE-like inhibitory protein
I*κ*B*α*: inhibitor of kappa B alpha
Mad: max-dimerization protein-1
E2F1: E2 promoter-binding factor-1
LRIG1: leucine-rich repeats and immunoglobulin-like domain-1
IRF1: interferon regulatory factor-1
USP11: ubiquitin specific peptidase 11.

## References

[bib1] Hanahan D , Weinberg RA . The hallmarks of cancer. Cell 2000; 100: 57–70. 10647931 10.1016/s0092-8674(00)81683-9

[bib2] Hanahan D , Weinberg RA . Hallmarks of cancer: the next generation. Cell 2011; 144: 646–674. 21376230 10.1016/j.cell.2011.02.013

[bib3] Galluzzi L , Vitale I , Abrams JM , Alnemri ES , Baehrecke EH , Blagosklonny MV et al. Molecular definitions of cell death subroutines: recommendations of the Nomenclature Committee on Cell Death 2012. Cell Death Differ 2012; 19: 107–120. 21760595 10.1038/cdd.2011.96 PMC3252826

[bib4] Galluzzi L , Bravo-San Pedro JM , Kroemer G . Organelle-specific initiation of cell death. Nat Cell Biol 2014; 16: 728–736. 25082195 10.1038/ncb3005

[bib5] Fulda S , Debatin KM . Extrinsic versus intrinsic apoptosis pathways in anticancer chemotherapy. Oncogene 2006; 25: 4798–4811. 16892092 10.1038/sj.onc.1209608

[bib6] Schutze S , Tchikov V , Schneider-Brachert W . Regulation of TNFR1 and CD95 signalling by receptor compartmentalization. Nat Rev Mol Cell Biol 2008; 9: 655–662. 18545270 10.1038/nrm2430

[bib7] Tait SW , Green DR . Mitochondria and cell death: outer membrane permeabilization and beyond. Nat Rev Mol Cell Biol 2010; 11: 621–632. 20683470 10.1038/nrm2952

[bib8] Fulda S , Vucic D . Targeting IAP proteins for therapeutic intervention in cancer. Nat Rev Drug Discov 2012; 11: 109–124. 22293567 10.1038/nrd3627

[bib9] Mahoney DJ , Cheung HH , Mrad RL , Plenchette S , Simard C , Enwere E et al. Both cIAP1 and cIAP2 regulate TNFalpha-mediated NF-kappaB activation. Proc Natl Acad Sci USA 2008; 105: 11778–11783. 18697935 10.1073/pnas.0711122105 PMC2575330

[bib10] Karin M . Nuclear factor-kappaB in cancer development and progression. Nature 2006; 441: 431–436. 16724054 10.1038/nature04870

[bib11] Micheau O , Tschopp J . Induction of TNF receptor I-mediated apoptosis via two sequential signaling complexes. Cell 2003; 114: 181–190. 12887920 10.1016/s0092-8674(03)00521-x

[bib12] Declercq W , Vanden Berghe T , Vandenabeele P . RIP kinases at the crossroads of cell death and survival. Cell 2009; 138: 229–232. 19632174 10.1016/j.cell.2009.07.006

[bib13] Eschenburg G , Eggert A , Schramm A , Lode HN , Hundsdoerfer P . Smac mimetic LBW242 sensitizes XIAP-overexpressing neuroblastoma cells for TNF-alpha-independent apoptosis. Cancer Res 2012; 72: 2645–2656. 22491673 10.1158/0008-5472.CAN-11-4072

[bib14] Allensworth JL , Sauer SJ , Lyerly HK , Morse MA , Devi GR . Smac mimetic Birinapant induces apoptosis and enhances TRAIL potency in inflammatory breast cancer cells in an IAP-dependent and TNF-alpha-independent mechanism. Breast Cancer Res Treat 2013; 137: 359–371. 23225169 10.1007/s10549-012-2352-6

[bib15] Petersen SL , Wang L , Yalcin-Chin A , Li L , Peyton M , Minna J et al. Autocrine TNFalpha signaling renders human cancer cells susceptible to Smac-mimetic-induced apoptosis. Cancer Cell 2007; 12: 445–456. 17996648 10.1016/j.ccr.2007.08.029 PMC3431210

[bib16] Varfolomeev E , Blankenship JW , Wayson SM , Fedorova AV , Kayagaki N , Garg P et al. IAP antagonists induce autoubiquitination of c-IAPs, NF-kappaB activation, and TNFalpha-dependent apoptosis. Cell 2007; 131: 669–681. 18022362 10.1016/j.cell.2007.10.030

[bib17] Vince JE , Wong WW , Khan N , Feltham R , Chau D , Ahmed AU et al. IAP antagonists target cIAP1 to induce TNFalpha-dependent apoptosis. Cell 2007; 131: 682–693. 18022363 10.1016/j.cell.2007.10.037

[bib18] Zarnegar BJ , Wang Y , Mahoney DJ , Dempsey PW , Cheung HH , He J et al. Noncanonical NF-kappaB activation requires coordinated assembly of a regulatory complex of the adaptors cIAP1, cIAP2, TRAF2 and TRAF3 and the kinase NIK. Nat Immunol 2008; 9: 1371–1378. 18997794 10.1038/ni.1676 PMC2676931

[bib19] Vallabhapurapu S , Matsuzawa A , Zhang W , Tseng PH , Keats JJ , Wang H et al. Nonredundant and complementary functions of TRAF2 and TRAF3 in a ubiquitination cascade that activates NIK-dependent alternative NF-kappaB signaling. Nat Immunol 2008; 9: 1364–1370. 18997792 10.1038/ni.1678 PMC2671996

[bib20] Masoumi KC , Cornmark L , Lonne GK , Hellman U , Larsson C . Identification of a novel protein kinase Cdelta-Smac complex that dissociates during paclitaxel-induced cell death. FEBS Lett 2012; 586: 1166–1172. 22465666 10.1016/j.febslet.2012.03.033

[bib21] Tenev T , Bianchi K , Darding M , Broemer M , Langlais C , Wallberg F et al. The Ripoptosome, a signaling platform that assembles in response to genotoxic stress and loss of IAPs. Mol Cell 2011; 43: 432–448. 21737329 10.1016/j.molcel.2011.06.006

[bib22] Young CY , Murtha PE , Zhang J . Tumor-promoting phorbol ester-induced cell death and gene expression in a human prostate adenocarcinoma cell line. Oncol Res 1994; 6: 203–210. 7841543

[bib23] Hernandez-Vargas H , Rodriguez-Pinilla SM , Julian-Tendero M , Sanchez-Rovira P , Cuevas C , Anton A et al. Gene expression profiling of breast cancer cells in response to gemcitabine: NF-kappaB pathway activation as a potential mechanism of resistance. Breast Cancer Res Treat 2007; 102: 157–172. 17039268 10.1007/s10549-006-9322-9

[bib24] Greer RM , Peyton M , Larsen JE , Girard L , Xie Y , Gazdar AF et al. SMAC mimetic (JP1201) sensitizes non-small cell lung cancers to multiple chemotherapy agents in an IAP-dependent but TNF-alpha-independent manner. Cancer Res 2011; 71: 7640–7648. 22049529 10.1158/0008-5472.CAN-10-3947 PMC3382117

[bib25] Beug ST , Tang VA , LaCasse EC , Cheung HH , Beauregard CE , Brun J et al. Smac mimetics and innate immune stimuli synergize to promote tumor death. Nat Biotechnol 2014; 32: 182–190. 24463573 10.1038/nbt.2806 PMC5030098

[bib26] Baeuerle PA , Baltimore D . Activation of DNA-binding activity in an apparently cytoplasmic precursor of the NF-kappa B transcription factor. Cell 1988; 53: 211–217. 3129195 10.1016/0092-8674(88)90382-0

[bib27] Holden NS , Squires PE , Kaur M , Bland R , Jones CE , Newton R . Phorbol ester-stimulated NF-kappaB-dependent transcription: roles for isoforms of novel protein kinase C. Cell Signal 2008; 20: 1338–1348. 18436431 10.1016/j.cellsig.2008.03.001

[bib28] Sen R , Baltimore D . Inducibility of kappa immunoglobulin enhancer-binding protein Nf-kappa B by a posttranslational mechanism. Cell 1986; 47: 921–928. 3096580 10.1016/0092-8674(86)90807-x

[bib29] Garg R , Blando J , Perez CJ , Wang H , Benavides FJ , Kazanietz MG . Activation of nuclear factor kappaB (NF-kappaB) in prostate cancer is mediated by protein kinase C epsilon (PKCepsilon). J Biol Chem 2012; 287: 37570–37582. 22955280 10.1074/jbc.M112.398925 PMC3481351

[bib30] Crawford N , Stasik I , Holohan C , Majkut J , McGrath M , Johnston PG et al. SAHA overcomes FLIP-mediated inhibition of SMAC mimetic-induced apoptosis in mesothelioma. Cell Death Dis 2013; 4: e733. 23868066 10.1038/cddis.2013.258 PMC3730428

[bib31] Pahl HL . Activators and target genes of Rel/NF-kappaB transcription factors. Oncogene 1999; 18: 6853–6866. 10602461 10.1038/sj.onc.1203239

[bib32] Tchoghandjian A , Jennewein C , Eckhardt I , Rajalingam K , Fulda S . Identification of non-canonical NF-kappaB signaling as a critical mediator of Smac mimetic-stimulated migration and invasion of glioblastoma cells. Cell Death Dis 2013; 4: e564. 23538445 10.1038/cddis.2013.70 PMC3615728

[bib33] Wagner L , Marschall V , Karl S , Cristofanon S , Zobel K , Deshayes K et al. Smac mimetic sensitizes glioblastoma cells to temozolomide-induced apoptosis in a RIP1- and NF-kappaB-dependent manner. Oncogene 2013; 32: 988–997. 22469979 10.1038/onc.2012.108

[bib34] Xu L , Zhu J , Hu X , Zhu H , Kim HT , LaBaer J et al. c-IAP1 cooperates with Myc by acting as a ubiquitin ligase for Mad1. Mol Cell 2007; 28: 914–922. 18082613 10.1016/j.molcel.2007.10.027

[bib35] Cartier J , Berthelet J , Marivin A , Gemble S , Edmond V , Plenchette S et al. Cellular inhibitor of apoptosis protein-1 (cIAP1) can regulate E2F1 transcription factor-mediated control of cyclin transcription. J Biol Chem 2011; 286: 26406–26417. 21653699 10.1074/jbc.M110.191239 PMC3143604

[bib36] Oberoi TK , Dogan T , Hocking JC , Scholz RP , Mooz J , Anderson CL et al. IAPs regulate the plasticity of cell migration by directly targeting Rac1 for degradation. EMBO J 2012; 31: 14–28. 22117219 10.1038/emboj.2011.423 PMC3252583

[bib37] Marivin A , Berthelet J , Cartier J , Paul C , Gemble S , Morizot A et al. cIAP1 regulates TNF-mediated cdc42 activation and filopodia formation. Oncogene 2014; 33: 5534–5545. 24276241 10.1038/onc.2013.499

[bib38] Bai L , McEachern D , Yang CY , Lu J , Sun H , Wang S . LRIG1 modulates cancer cell sensitivity to Smac mimetics by regulating TNFalpha expression and receptor tyrosine kinase signaling. Cancer Res 2012; 72: 1229–1238. 22241084 10.1158/0008-5472.CAN-11-2428 PMC3294058

[bib39] Eckhardt I , Weigert A , Fulda S . Identification of IRF1 as critical dual regulator of Smac mimetic-induced apoptosis and inflammatory cytokine response. Cell Death Dis 2014; 5: e1562. 25501823 10.1038/cddis.2014.498 PMC4454156

[bib40] Lee EW , Seong D , Seo J , Jeong M , Lee HK , Song J . USP11-dependent selective cIAP2 deubiquitylation and stabilization determine sensitivity to Smac mimetics. Cell Death Differ 2015; 22: 1463–1476. 25613375 10.1038/cdd.2014.234 PMC4532773

[bib41] Lofstedt T , Jogi A , Sigvardsson M , Gradin K , Poellinger L , Pahlman S et al. Induction of ID2 expression by hypoxia-inducible factor-1: a role in dedifferentiation of hypoxic neuroblastoma cells. J Biol Chem 2004; 279: 39223–39231. 15252039 10.1074/jbc.M402904200

[bib42] De Preter K , Speleman F , Combaret V , Lunec J , Laureys G , Eussen BH et al. Quantification of MYCN, DDX1, and NAG gene copy number in neuroblastoma using a real-time quantitative PCR assay. Mod Pathol 2002; 15: 159–166. 11850545 10.1038/modpathol.3880508

[bib43] Kasperczyk H , La Ferla-Bruhl K , Westhoff MA , Behrend L , Zwacka RM , Debatin KM et al. Betulinic acid as new activator of NF-kappaB: molecular mechanisms and implications for cancer therapy. Oncogene 2005; 24: 6945–6956. 16007147 10.1038/sj.onc.1208842

[bib44] Tusher VG , Tibshirani R , Chu G . Significance analysis of microarrays applied to the ionizing radiation response. Proc Natl Acad Sci USA 2001; 98: 5116–5121. 11309499 10.1073/pnas.091062498 PMC33173

