# Supplementary figures and images for: PKC activation sensitizes basal-like breast cancer cell lines to Smac mimetics
Source: Cell Death Discov. 2016 Feb 29;2:16002–. doi: 10.1038/cddiscovery.2016.2 (PMC4979953; doi:10.1038/cddiscovery.2016.2)

# Supplementary Figure 1

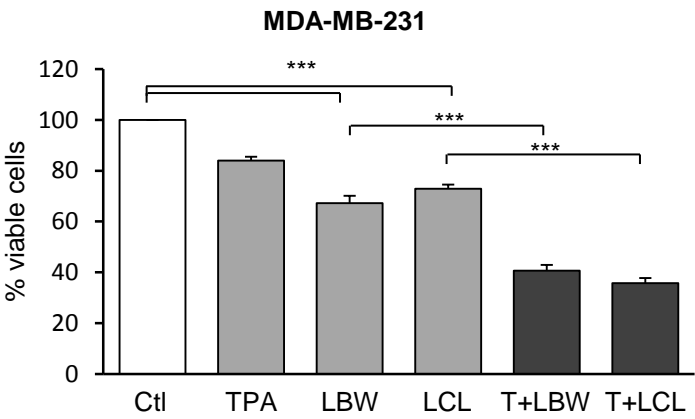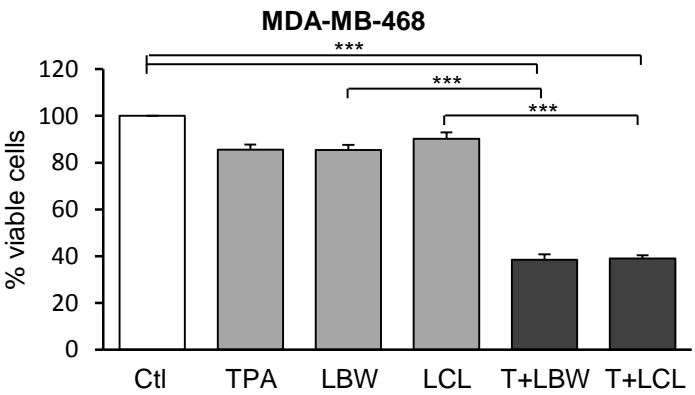

Supplement: Supplementary Figure 1 [file cddiscovery20162-s1.pdf]

# Supplementary Figure 2

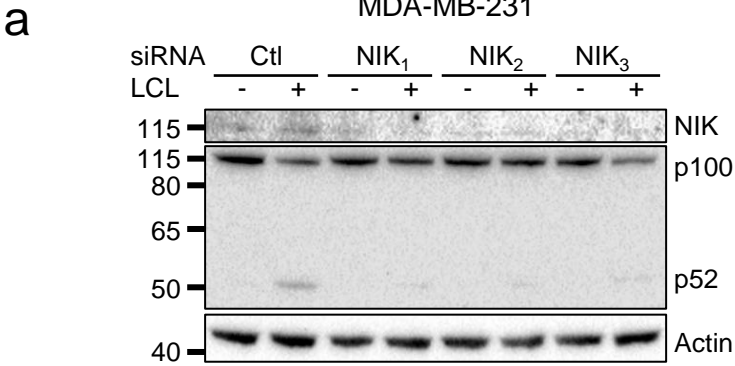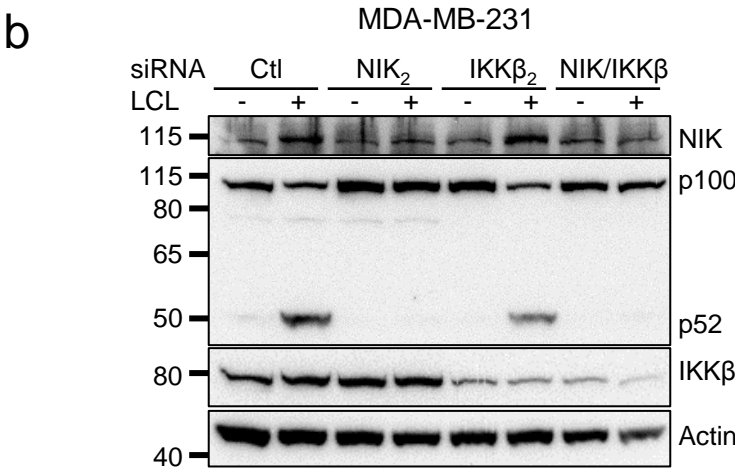

Supplement: Supplementary Figure 2 [file cddiscovery20162-s2.pdf]

# Supplementary Figure 3

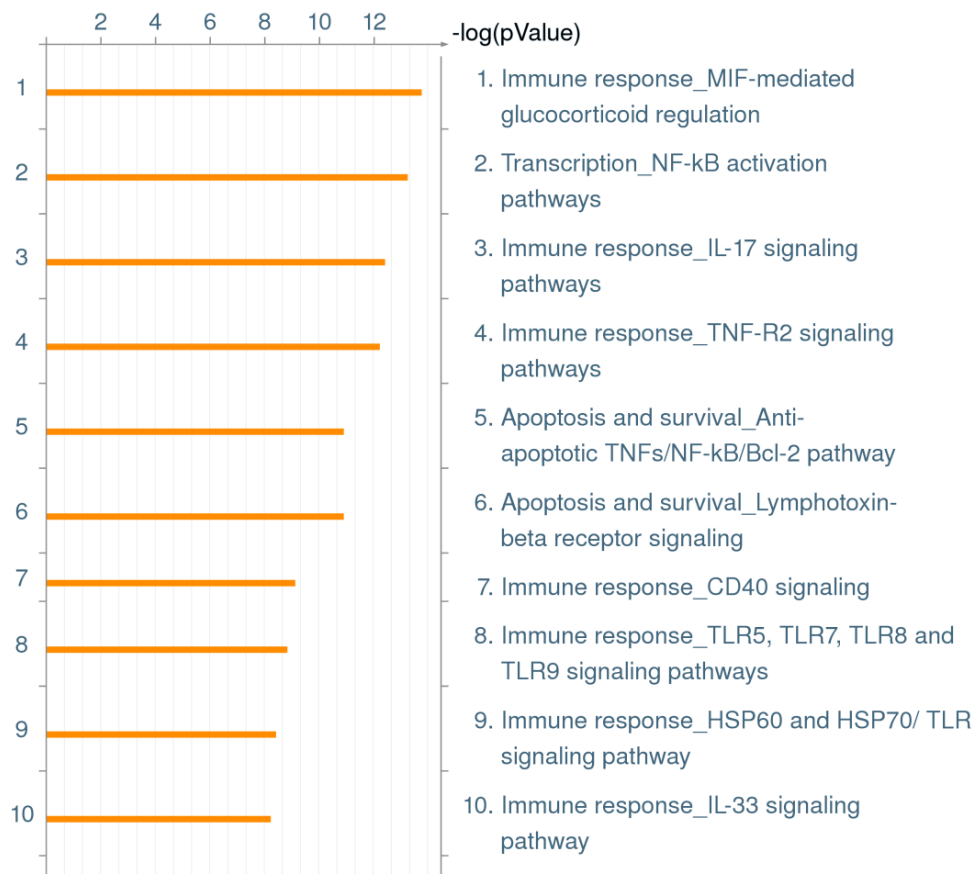

Supplement: Supplementary Figure 3 [file cddiscovery20162-s3.pdf]
