# Supplementary material for: PKC activation sensitizes basal-like breast cancer cell lines to Smac mimetics
Source: Cell Death Discov. 2016 Feb 29;2:16002–. doi: 10.1038/cddiscovery.2016.2 (PMC4979953; doi:10.1038/cddiscovery.2016.2)
Supplement: Supplementary Table 1 [file cddiscovery20162-s4.pdf]

MDA-MB-231

ILMN ID	gene symbol	FC	q-value (%)	ILMN ID	gene symbol	FC	q-value (%)
ILMN_1776181	BIRC3	4.133616207	0	ILMN_3243644	LOC100132564	1.488056285	0
ILMN_2171789	TRAF1	4.022371124	0	ILMN_1654072	CX3CL1	1.482551024	0
ILMN_3308138	RNU4-2	3.560636548	0	ILMN_1657996	LOC642035	1.470678497	0
ILMN_1682636	CXCL2	3.338696132	0	ILMN_1727689	TNFAIP2	1.470391759	0
ILMN_1699651	IL6	2.885870253	0	ILMN_2137789	KLF4	1.459177507	0
ILMN_2184373	IL8	2.672851583	0	ILMN_1659936	PPP1R15A	1.459055361	0
ILMN_1691846	G0S2	2.588732346	0	ILMN_3249286	SNORD12C	1.456585254	0
ILMN_1773154	NFKBIA	2.586283034	0	ILMN_2189027	LIPG	1.45232032	0
ILMN_1717313	NFKBIE	2.578580764	0	ILMN_1706645	C6orf150	1.447531969	0
ILMN_1787897	CXCL1	2.366912402	0	ILMN_1666986	GCNT2	1.442174217	0
ILMN_1797728	HMGCS1	2.161407612	0	ILMN_1666893	FLJ25801	1.439009882	0
ILMN_3249501	ZNF697	2.060884838	0	ILMN_3244646	RNU1G2	1.436584243	0
ILMN_1702691	TNFAIP3	2.058522239	0	ILMN_1672295	ZC3H12A	1.432795066	0
ILMN_1745964	IRAK2	2.05514163	0	ILMN_1721316	TNFRSF10A	1.422661138	0
ILMN_1739423	RN7SK	2.044089637	0	ILMN_1674985	TMEM51	1.420488783	0
ILMN_1762899	EGR1	2.026076817	0	ILMN_2331010	TNFRSF10B	1.418159752	0
ILMN_2052208	GADD45A	2.017179931	0	ILMN_1737406	KLF6	1.41262379	0
ILMN_1789793	NUAK2	2.010445018	0	ILMN_1782305	NR4A2	1.408071808	0
ILMN_2186137	RRAD	1.974343562	0	ILMN_1682775	EDN1	1.406126216	0
ILMN_2121408	HBEGF	1.964702195	0	ILMN_1686454	TIFA	1.405810981	0
ILMN_2374865	ATF3	1.935953935	0	ILMN_1805543	ADAMTS9	1.401272278	0
ILMN_1712708	TRIM47	1.904057585	0	ILMN_2389064	C15orf48	1.398474351	0
ILMN_1724070	MAP3K14	1.875666001	0	ILMN_3240594	RNU4ATAC	1.397125654	0
ILMN_1710514	BCL3	1.872116619	0	ILMN_2053415	LDLR	1.396798107	0
ILMN_1682081	IBRDC3	1.855950499	0	ILMN_1680054	LAMB3	1.392861159	0
ILMN_1795930	PTGER4	1.841437173	0	ILMN_2401873	DUSP10	1.392212698	0
ILMN_2390859	NFKB2	1.836548457	0	ILMN_1775743	BTG1	1.391697965	0
ILMN_1781285	DUSP1	1.810732946	0	ILMN_1770260	NFKBIZ	1.390344905	0
ILMN_1665838	SLC25A24	1.78612838	0	ILMN_1668850	PCSK9	1.388660378	0
ILMN_1709044	TGIF2	1.741618026	0	ILMN_2110908	MYC	1.387756094	0
ILMN_2086077	JUNB	1.699866483	0	ILMN_1689842	SC4MOL	1.380774893	0
ILMN_1669523	FOS	1.695085657	0	ILMN_1780582	CD83	1.377134034	0
ILMN_1652777	CDC42EP2	1.683091767	0	ILMN_1782050	CEBPD	1.374133839	0
ILMN_1656501	DUSP5	1.682904723	0	ILMN_1693014	CEBPB	1.370978812	0
ILMN_1758938	SLC31A2	1.6514344	0	ILMN_1699354	EPHA2	1.36788415	0
ILMN_1812226	ICAM1	1.631331375	0	ILMN_2376204	LTB	1.367616122	0
ILMN_1657395	HMGCR	1.629686146	0	ILMN_1652082	ELF4	1.364178363	0
ILMN_1793474	INSIG1	1.628186531	0	ILMN_1738725	LIF	1.358790716	0
ILMN_1735231	ZSWIM4	1.605257385	0	ILMN_1704055	HSPC111	1.351889186	0
ILMN_1698404	ERN1	1.596020483	0	ILMN_1735548	HIVEP1	1.347934319	0
ILMN_1717706	PLK2	1.581699317	0	ILMN_1813704	KIAA1199	1.341097781	0
ILMN_3247139	C17orf96	1.56862536	0	ILMN_1703123	AXUD1	1.337571156	0
ILMN_3246273	RNU1-3	1.553065009	0	ILMN_3309453	RNU4-1	1.330571197	0
ILMN_1811258	RELB	1.546518208	0	ILMN_1792910	MNT	1.329125204	0
ILMN_1768534	BHLHB2	1.544607011	0	ILMN_1661197	CLCF1	1.324915175	0
ILMN_1689378	CCRN4L	1.53885052	0	ILMN_1697817	PANX1	1.315182281	0
ILMN_3237966	FBXL11	1.533803356	0	ILMN_1733627	NEDD4L	1.313762281	0
ILMN_3236653	RNU1-5	1.52515842	0	ILMN_1724686	CLDN1	1.304274167	0
ILMN_1728106	TNF	1.521855856	0	ILMN_1703650	TNIP1	1.299025837	0
ILMN_1808811	SBNO2	1.519615218	0	ILMN_1751464	TNFSF9	1.295130708	0
ILMN_1707748	PIM3	1.509777395	0	ILMN_1656186	SLC41A1	1.277875171	0
ILMN_1687538	ETS1	1.502254677	0	ILMN_2128795	LRIG1	1.268015973	0
ILMN_1657234	CCL20	1.499614809	0	ILMN_1702487	SGK	1.250008117	0
ILMN_1708375	IRF1	1.494438954	0	ILMN_2197128	OSR1	-1.876282613	0

MDA-MB-468

ILMN ID	gene symbol	FC	q-value (%)
ILMN_1776181	BIRC3	2.646435096	0
ILMN_1767556	C10orf10	1.736967105	0
ILMN_1787567	TSC22D1	1.650963224	0
ILMN_1717313	NFKBIE	1.512398074	0
ILMN_2053415	LDLR	1.432746401	0
ILMN_2049766	NFE2L3	1.42886733	0
ILMN_1671554	LPIN1	1.412024473	0
ILMN_1773154	NFKBIA	1.401994962	0
ILMN_1763036	CLCN6	1.253676407	0

Supplementary Table I
