# Supplementary material for: PKC activation sensitizes basal-like breast cancer cell lines to Smac mimetics
Source: Cell Death Discov. 2016 Feb 29;2:16002–. doi: 10.1038/cddiscovery.2016.2 (PMC4979953; doi:10.1038/cddiscovery.2016.2)
Supplement: Supplementary Information [file cddiscovery20162-s5.doc]

**Supplementary Table 1**

Significance analysis of microarrays (SAM) analysis of gene expression in control and SM-treated MDA-MB-231 (top) and MDA-MB-468 (bottom) cells. Genes with a q-value of 0% are included and sorted according to their fold change (FC) in log2 expression. Data are from three independent experiments.

**Supplementary figure 1**

MDA-MB-231 and MDA-MB-468 cells were treated with 16 nM TPA, 20 µM LBW242, 10 µM LCL161, alone or with the combinations TPA+LBW242 or TPA+LCL161 for 30 h. The cell viability was measured with WST1. Graph represents the mean ± SEM of three independent experiments, *p <0.05, **p <0.01, ***p <0.001.

**Supplementary figure 2**

(**a**) MDA-MB-231 cells were transfected 48 h with three different siRNA oligos targeting NIK (siNIK1-3) and treated with or without 10 µM LCL161 for 3 h. NIK, p100 and p52. (**b**) MDA-MB-231 cells were transfected 48 h with siRNA oligos targeting NIK (NIK_2_), IKKβ (IKKβ_2_) or the combination of both (NIK/IKKβ) and treated with or without 10 µM LCL161 for 3 h. protein levels were analyzed with Western blot. Actin was used as a loading control.

**Supplementary figure 3**

Significantly differentially expressed genes in MDA-MB-231 cells from the SAM analysis were used in the Pathway analysis to reveal pathways that the differentially expressed genes upon SM treatment are involved in.
